# Scratch Resistance and Tribological Enhancement of Epoxy Composites Reinforced with Chopped Glass Fiber and Nano Silica Through Taguchi Analysis

**DOI:** 10.3390/polym17182550

**Published:** 2025-09-21

**Authors:** Elanur Ozun, Reyhan Ceylan, Mustafa Özgür Bora, Sinan Fidan, Satılmış Ürgün, Mehmet İskender Özsoy, Erman Güleç

**Affiliations:** 1 Institute of Science, Department of Mechanical Engineering, Kocaeli University, Kocaeli 41380, Turkey; elanurozun@gmail.com (E.O.); reyceylan@gmail.com (R.C.); 2 Faculty of Aeronautics and Astronautics, Department of Airframe & Powerplant Maintenance, Kocaeli University, Kocaeli 41001, Turkey; ozgur.bora@kocaeli.edu.tr (M.Ö.B.); sfidan@kocaeli.edu.tr (S.F.); 3 Faculty of Aeronautics and Astronautics, Department of Aviation Electrics and Electronics, Kocaeli University, Kocaeli 41001, Turkey; urgun@kocaeli.edu.tr; 4 Faculty of Engineering, Department of Mechanical Engineering, Sakarya University, Sakarya 54050, Turkey; 5 Otokar Automotive Defense Industry Corp., Sakarya 54580, Turkey; egulec@otokar.com.tr

**Keywords:** epoxy, glass fiber, nano-silica, scratch resistance, SEM analysis, ANOVA

## Abstract

This study examines the incorporation of chopped glass fiber and nano-silica into epoxy, focusing on their effects on the tribological and mechanical properties. Three reinforcement ratios (1 wt.%, 3 wt.%, and 5 wt.%) were analyzed by scratch tests and profilometric analysis. The coefficient of friction (COF), scratch depth, and scratch width values of the unreinforced epoxy resin were measured as 0.45, 37.73 µm and 479 µm, respectively. The addition of glass fibers contributed to improved scratch performance by restricting material removal and stabilizing groove morphology, although higher fiber ratios caused an increase in COF. The results indicated that nano-silica increased scratch resistance with a COF of 0.42 at 5 wt.%, giving a scratch depth of 19.92 µm and a scratch width of 166 µm. Glass fiber also improved scratch performance, although there were high COF values for higher ratios, which could be due to the aggregation effect of the fibers. Statistical validation of the results was carried out through the Taguchi method and ANOVA analyses. These analyses showed that reinforcement type and ratio played an important role in scratch behavior. SEM analyses of worn surfaces showed that nano-silica can dissipate stress and minimize plastic deformation to yield improved scratch morphology. Overall, the results emphasize the complementary role of glass fiber and nano-silica reinforcements in improving the scratch resistance of epoxy resin for industrial applications.

## 1. Introduction

As it is known, polymer materials which are mainly used in the production of composite materials stand out with their advantages such as low density, high dimensional stability, high thermal insulation, and relatively low processing costs [1,2,3,4,5,6,7]. However, in addition to the mechanical properties of polymers, their tribological behavior is gaining importance [8,9]. Regarding this, scratch tests define the tribological behavior of materials such as coefficient of friction (COF), scratch hardness, and critical normal load [10]. The sensitivity of polymeric materials to scratch is important not only for esthetic concerns but also for structural integrity and the functioning of the product [11,12,13]. Among polymer materials, thermosetting epoxy stands out with its various outstanding properties such as thermal stability, resistance to solvent environments, and easy machinability [14]. However, the relatively poor tribological properties of epoxy, compared to reinforced or modified systems, may limit its performance in certain industrial applications [15].

The addition of reinforcing materials into epoxy resins to improve their tribological behavior attracts the attention of researchers today. Synthetic reinforcement materials tend to affect the wear rate and COF values of polymer materials in a way that can be a complex process. In this context, the compatibility of the matrix material and the reinforcement material is one of the critical factors [16]. As reinforcement material, macro-sized fibers (glass, carbon, and aramid) or ceramic powders can be selected, as well as nano-sized particles, which are popular now. While nanoscale fillers are generally reported to enhance crack energy dissipation owing to their higher surface area and more effective stress transfer, microscale reinforcements can also contribute through mechanisms such as crack deflection and fiber pull-out. Therefore, the influence of filler size on energy dissipation is not absolute but depends on filler type, morphology, and interfacial bonding. In general, a higher number of nanoparticles may increase resistance to crack propagation compared to microscale fillers, although this effect can vary depending on the specific material system [17,18]. Traditional inorganic fillers such as silica (SiO_2_) or silicon carbide (SiC) are proven to effectively develop the wear resistance and mechanical performance of epoxy. Ceramics with superior thermal and wear performance have the ability to improve mechanical strength even with small reinforcement ratios [11]. With silica reinforcement, changes occur in the properties of the matrix material such as wear, thermal resistance, hardness, and modulus [16]. Glass fiber stands out with its extraordinary properties such as lightness, high strength, hardness, chemical resistance, and high temperature resistance. Despite these advantages, the effect of the matrix material on tribological performance is complicated, and the examination of the scratch behavior is of critical importance [19]. In this context, Ekrem et al. [20] added boron nitride nanoparticles to epoxy resin at 0.3, 0.5, 0.7, and 1 wt.% and determined the friction and wear behaviors of boron nitride reinforced epoxy resin using pin on disk testing. A decrease in COF and wear values was determined with boron nitride reinforcement in epoxy resin. While the COF value of unreinforced epoxy resin was 0.17, the COF values of epoxy resins reinforced with 0.3, 0.5, 0.7, and 1 wt.% boron nitride were 0.16, 0.7, 0.11, and 0.13, respectively. Wear rates also exhibited a similar trend with COF values, and the lowest wear rate was obtained in epoxy reinforced with 0.5 wt.% boron nitride (2.94 × 10^−10^ (mm^3^∙(Nm)^−1^)) compared to unreinforced epoxy (7.7 × 10^−10^ (mm^3^∙(Nm)^−1^)). In another study, Wang et al. [21] reinforced 0, 3, 6, 8, 10, and 15 vol.% nano-silica to epoxy resin in order to determine the effects of nano-silica reinforcement on the scratch behavior of epoxy resin. According to the scratch test results performed under different normal loads, the optimum reinforcement ratio for all situations was determined as 8 vol.% nano-silica. In this context, improvements of up to 9% and 25% in COF value and scratch depth at 8 vol.% silica reinforcement were achieved, respectively, compared to the unreinforced epoxy. Zhao et al. reinforced epoxy with 5, 10, and 15 wt.% short glass fiber and evaluated the scratch performance of epoxy under oil lubrication conditions. When the scratch test results were evaluated, positive improvements in scratch performance were recorded for all reinforcement ratios. The lowest COF values were achieved with 5 wt.% short glass fiber reinforcement, with a 77% improvement compared to unreinforced epoxy. The COF values of epoxy with 10 wt.% and 15 wt.% short glass fiber reinforcement were observed to decrease by 67% and 27%, respectively, compared to unreinforced epoxy [22]. On the other hand, Ozcan et al. [23] added 1 wt.% nano-silica and 0.1 wt.% graphene into epoxy resin and performed scratch tests with a diamond Rockwell indenter at a normal load of 0.05–5 N. The penetration depths of the silica and graphene reinforced samples decreased by approximately 10% and 12%, respectively, compared to unreinforced epoxy. In addition, the microhardness values increased by 3.84% and 7.69%, respectively, in the silica and graphene reinforced samples compared to unreinforced epoxy.

From an industrial perspective, the tribological performance of materials is an important issue, especially in parts that move relatively. Nowadays, the tribological performance of polymeric based materials, which dominate many leading sectors, needs to be improved. With this goal, reinforcing various materials into polymeric materials has become one of the research topics. In the literature studies examined, it was determined that the parameters such as the type, size, shape, and reinforcement ratio of the reinforcement material significantly affect the tribological performance of epoxy resin. However, it was determined that there are a limited number of studies in the literature examining the effects of these parameters on the scratching behavior of epoxy. Many different parameters affect the scratch test results, and statistical approaches provide a more accurate evaluation of the results. It was determined that no statistical method was used to support the experimental results in the reviewed literature studies. Also, the Taguchi method, which was applied to determine the most effective parameters and to create experimental sets in this context, was not encountered in the literature studies. Additionally, it was found that the effect of nano- and micro-sized reinforcement materials on the scratch mechanism was not investigated in detail. Moreover, the effect of reinforcement materials on tribological performance is a complicated phenomenon, and it is necessary to examine their effects on mechanical performance simultaneously. In this context, in this study, chopped glass fiber and nano-silica were separately reinforced into epoxy resin at different reinforcement ratios (1 wt.%, 3 wt.%, and 5 wt.%). After performing scratch tests and profilometer measurements, the performances of epoxy resin reinforced with chopped glass fiber and nano-silica at different weight ratios were evaluated compared to unreinforced epoxy resin. In addition, Design of Experiments (DOE) was performed through Taguchi method to increase efficiency by systematically examining the effects of multiple variables. Statistical analysis (ANOVA) method was used to support the experimental results and evaluate them more accurately. Finally, SEM images were taken, and morphological analysis was performed in order to perform detailed damage analysis of the worn surfaces.

## 2. Materials and Methods

### 2.1. Materials

In this study, two-component Araldite 2015 was used as epoxy and was supplied by Dost Kimya, Istanbul, Turkey. The technical properties of the epoxy resin, which stands out with its high performance in composite applications, were given in Table 1 according to supplier company data. Chopped glass fiber and nano-silica were added as reinforcement materials into the epoxy resin. Glass fibers are 3 mm long and 13–15 µm in diameter and were supplied by Dost Kimya, Istanbul, Turkey. Silicon dioxide has a particle size of 18–35 nm, a purity of 96.3%, a specific surface area of 150–550 m^2^ g^−1^, exhibits hydrophilic surface characteristics, and was supplied by Nanografi Nanotechnology, Ankara, Turkey. The chopped glass fibers and nano-silica particles were coated with a silane-based sizing agent, which improves adhesion and ensures strong interfacial bonding with the epoxy matrix. In addition, the nano-silica particles are hydrophilic.

### 2.2. Preparation of the Samples

**Figure 1:**
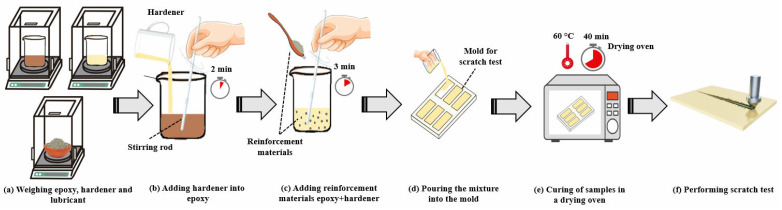
Preparation stages of the samples.

The epoxy was prepared in accordance with its technical datasheet, ensuring the epoxy resin to hardener ratio was maintained at 100:100 by weight. The epoxy and hardener were precisely measured using a Weightlab WH-503T precision scale, which has an accuracy of 0.001 g. The mixing process was conducted manually with a spoon at room temperature for a duration of 2 min. Subsequently, various weight percentages (1, 3, and 5 wt.%) of chopped glass fiber and nano-silica were added to the mixture. The reinforced epoxy resin mixture was mixed manually using a spoon for 3 min. The resultant mixture of epoxy, reinforcement materials, and hardener was subsequently poured into Teflon mold, previously treated with mold release spray. Curing was executed in an oven at 60 °C for 40 min, in accordance with the specifications outlined in the technical datasheet. After the initial curing stage, the Teflon mold was transferred to a different oven maintained at room temperature for a normalization period of 24 h, ensuring complete curing of the samples. Upon completion of the curing process, the samples were removed from the Teflon mold. Afterwards, the sample surfaces were polished to remove any irregularities and ensure a smooth, uniform surface suitable for scratch testing. The preparation stages of the samples are shown schematically in Figure 1.

### 2.3. Performing the Scratch Tests

Scratch tests were conducted using the “CSM Micro Scratch Test Device” at TUBITAK-MAM in Kocaeli, Turkey, to characterize the scratch resistance of reinforced epoxy samples, in accordance with the ASTM D7027-13 [24] standard. During the test, samples were subjected to scratching using a Rockwell indenter with a 50 μm radius spherical diamond. To minimize surface roughness, preliminary scanning of the sample surfaces was performed using a small load of approximately 0.03 N. Subsequently, scratch tests were executed by applying a load of 15 N to the sample surface at a speed of 30 mm/min over a distance of 10 mm. Each sample underwent three replicates, and the mean values were utilized for analysis.

### 2.4. Profilometer Roughness Analysis

Throughout the roughness test, Nanovea PS50 non-contact laser profilometer was used in measuring the surface roughness of chopped glass fiber reinforced epoxy, nano-silica reinforced epoxy, and unreinforced epoxy samples. This was then used in viewing topography and shapes of the samples. The profilometry scans were set with a scan distance of 1 mm on the X-axis and steps of 20 µm. The Y-axis scan was set over a 12 mm distance, using increased steps of 30 µm. Scan velocity remained at 20 mm/s, given the best compromise between accuracy of data and efficiency of operations. The sensor settings can also be altered to output high-resolution topographic data in 1000 Hz. This result is in accordance with ISO 25178 [25] for the measurement of areal surface texture parameters and ISO 4287 [26] for the determination of line roughness. Following these standards, there has been an accurate capture of surface parameters, such as Sa and Ra, to allow standardized evaluation of roughness and texture features. Measurement accuracy has, therefore, been important in assessing the influence of reinforcement types and ratios on the surface characteristics in epoxy.

**Figure 2:**
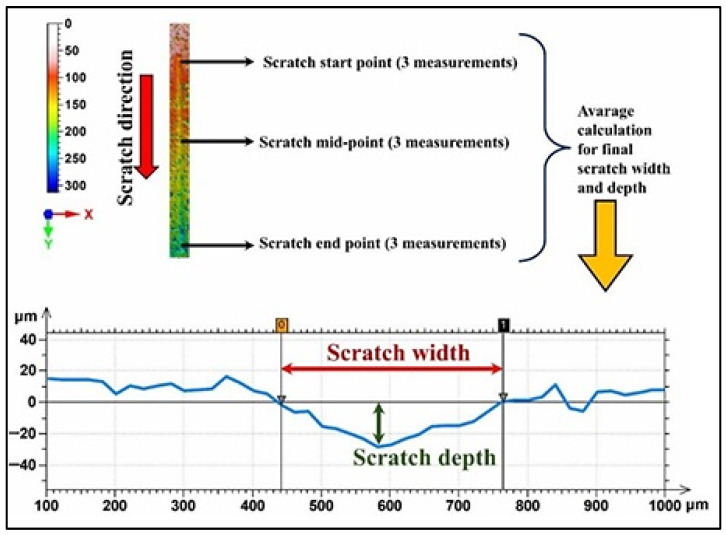
Scratch width and depth measurement methodology with profilometric measurement approach.

The profilometric measurement method is shown in Figure 2 for the calculation of scratch width and scratch depth in the material samples. To achieve accurate and representative data, three measurements were performed on each scratch path at three particular locations: scratch start point, scratch middle point, and scratch end point. In all of these cases, three independent measurements were made to capture the variability in scratch dimensions and further ensure the reliability of the obtained data. All profilometric measurements involved the following: a depth profile across the scratch width at each location was recorded. Scratch width is defined as the horizontal spacing between the two sides of the scratch wherein the surface level deviates significantly from the base level. It also included scratch depth, which was defined as the maximum vertical distance from the surface baseline to the lowest point within the scratch track and the surface material depth displaced by scratching. Data was collected at the start, midpoint, and endpoint, after which the average calculation was performed for both scratch width and scratch depth. Scratch depth denotes the mean of the three baseline-leveled peak depths taken from cross-sections at the start, middle, and end of each scratch. Furthermore, the full depth distribution along the track and its maximum values were also considered. This averaging method minimizes the local variations in scratch resistance along the scratch path and gives a value more representative of the general characteristics of the scratch. Further analysis was thus derived from these values when comparing the scratch resistance of the various epoxy resins which were reinforced by different types at different percentages of reinforcement. To mitigate artificial extremes, saturated or invalid height samples (instrument clip flags), and obvious chip-out spikes were excluded from descriptive statistics, and group comparisons were based on median, interquartile range, and percentiles rather than maxima. The measurement methodology here follows standardized practices concerning the assessment of the surface wear and deformation in reinforced polymers.

### 2.5. Analysis of Variance Analyses (ANOVA)

Analysis of variance (ANOVA) was used in this study to assess the statistical significance of the main factors (material type and reinforcement ratio) and their interaction effects on the performance measures of epoxy. ANOVA is highly advantageous as it allows the identification of both individual and interactive contributions of factors to the overall performance. The factorial experimental design can systematically vary the levels of each factor and helps us to provide insight into how changes in glass fiber and nano-silica content affect the mechanical properties of the epoxy. An important benefit of the factorial design approach is that it reduces the number of experiments required to investigate the effect of multiple variables simultaneously, thus saving time and resources. It also provides a better understanding of the interaction between factors, which is very important in material science, where the combined effects of supplements may be synergistic or even antagonistic, not just additive. ANOVA also provides clear information about variability in the data, allowing us to determine whether observed differences in performance measures are statistically significant or are due to random variation. The use of ANOVA in this study ensures that conclusions drawn about supplement effects are robust and scientifically valid.

In this study, a general factorial regression method was used to analyze the effect of glass fiber and nano-silica reinforcements on the performance of epoxy resin. The experimental design included six formulations with varying reinforcement concentrations (1%, 3%, and 5% by weight) for both glass fiber and nano-silica. The choice of these concentrations was based on previous research in the literature, which emphasizes that these ratios are optimal for improving the mechanical properties of epoxy composites.

### 2.6. Scanning Electron Microscopy (SEM) Analyses

To examine the fracture surfaces in detail after scratch tests, the sample surfaces were gold-coated with the Cressington Sputter Coater 108 Auto device, and then SEM (Scanning Electron Microscopy) images were taken with 100× magnification by the Tescan Vega3 device.

## 3. Results

### 3.1. The Variation in COF Values

**Figure 3:**
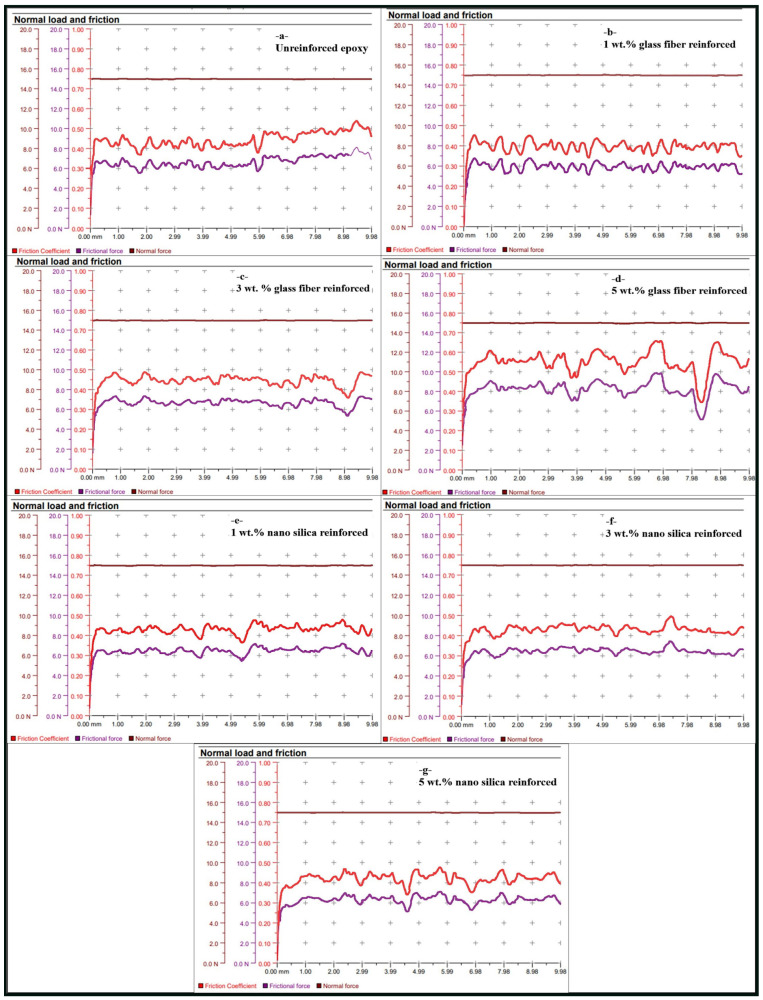
COF values of epoxy: (**a**) unreinforced, (**b**) 1 wt.% glass fiber reinforced, (**c**) 3 wt.% glass fiber reinforced, (**d**) 5 wt.% glass fiber reinforced, (**e**) 1 wt.% nano-silica reinforced, (**f**) 3 wt.% nano-silica reinforced, and (**g**) 5 wt.% nano-silica reinforced.

For both unreinforced and glass fiber and nano-silica reinforced epoxy, the change in COF during scratch testing is examined. As expected, the friction force curve exhibits the same trend as the COF curve, since the applied normal load was kept constant throughout the tests. The elasticity and surface properties of the polymer are the only factors that affect the COF in the absence of any additives, resulting in a value of 0.45 for unreinforced epoxy. When compared to unreinforced epoxy (Figure 3a), a little decrease in the COF (0.44) was seen at 1 wt.% chopped glass fiber (Figure 3b). Small quantities of stiff glass fibers are introduced, and this causes localized hardening, which lowers the friction brought on by surface deformation under scratch load. An increase in the COF (0.46) occurred at 3 wt.% chopped glass fiber content (Figure 3c). As a result of the improved surface hardness and scratch resistance brought about by the higher fiber content (Figure 3d), the sample was less susceptible to wear and surface deformation, which normally lowers the COF. Since fiber aggregation, the COF (0.57) increased at 5 wt.% glass fiber as opposed to 3 wt.%, resulting in greater abrasive fiber contact with the scratch indenter. Frictional resistance during the test may rise due to extra surface contact caused by the stiff glass fibers. And also, the addition of 1 wt.% of nano-silica (Figure 3e) tends to lower the COF (due to its capacity to fill in the gaps in the polymer matrix, improving the hardness and smoothness of the surface. A more significant reduction in the coefficient of friction (COF) is expected for 3 wt.% nano-silica, as the silica nanoparticles increase the stiffness of the matrix and reduce the material’s plastic deformation under scratch-induced strain (Figure 3f). The improved and more uniform load distribution facilitated by the nanoscale particles leads to reduced friction during testing. At 5 wt.% nano-silica, the COF slightly decreased to 0.42. This minor reduction is likely due to the nanoparticles helping to maintain a smoother surface, despite possible slight agglomeration at higher filler content.

As a result, at higher concentrations, filler agglomeration may occur, which can alter the frictional response. For glass fibers, this leads to increased surface roughness and a higher COF due to enhanced abrasive contacts during scratch testing, whereas for nano-silica, the COF slightly decreases as the nanoparticles help maintain surface smoothness despite partial agglomeration.

Schön et al. present that the behavior of friction force against grip displacement curve depends on the size of friction force [27]. When the latter is large, as often is the case, stick-slip phenomena are observed. In such cases, friction force increases linearly due to adhesion of the contact surfaces. This transition to sliding occurs when friction force suddenly drops. The displacement of grip also increases abruptly, simultaneously. Sometimes, this stick-slip behavior, without smooth transition, can start right at the time when the friction force just reaches its first high. Wear resistance of epoxy composites was evaluated on the basis of COF and specific wear rate [28]. With the rising of nanoparticle content, the COF values first decreased and then started to increase. At the 4 wt.% of TiO_2_, an optimum value had been achieved. The change in the COF varied from 0.10 to 0.06 and caused a decrease in fatigue resistance by unstable sliding of the nanoparticles when they were separated from the epoxy matrix at the interface of the friction pair. In good agreement, this already includes the microcutting and microdelamination parts of the resin wear process. Resin wear arises from fracturing of the bonding joints that result from the relative movement between contacting surfaces. Resin components of friction are controlled by the formation and rupture of the bonding junctions. While increased stiffness often correlates with higher frictional resistance, this relationship can be influenced by factors such as surface roughness, filler dispersion, and matrix-filler interactions. The important relationship existing between the mechanical properties, such as tensile modulus and friction behavior, may become imperative in applications where both strength and wear performance are required. This may mean that for applications where sliding contact is involved, the stiffer stronger composites require more effort to overcome friction. This might influence performance and design optimization aspects as well [29]. Temperature influences the relationship between sliding speed and friction coefficient, whereby, at increased sliding speeds, higher temperatures are generated. The friction coefficient exponentially rises with temperature as the polymer approaches its softening point. This is probably because a loss in structural integrity along with more distortion due to the polymer results in further resistance during sliding. Fillers, however, can reduce this effect. Moreover, at high temperatures, friction is reduced by the wear resistance and thermal stability of the polymer due to fillers. This is realized through the strengthening effect that the fillers have on the polymer matrix, thus keeping the friction coefficient lower under high-speed sliding conditions [30].

### 3.2. Evaluation of Profilometer Analyses

**Figure 4:**
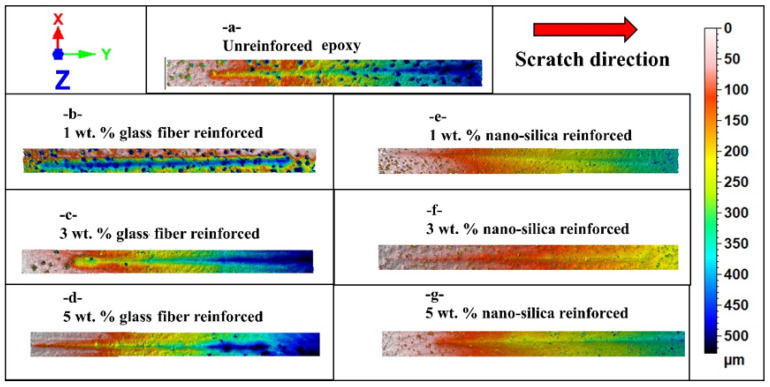
Surface morphologies comparison of epoxy: (**a**) unreinforced, (**b**) 1 wt.% glass fiber rein forced, (**c**) 3 wt.% glass fiber reinforced, (**d**) 5 wt.% glass fiber reinforced, (**e**) 1 wt.% nano-silica rein forced, (**f**) 3 wt.% nano-silica reinforced, and (**g**) 5 wt.% nano-silica reinforced.

Figure 4 represents the morphological variation in scratch track for unreinforced epoxy and epoxy reinforced with glass fiber and nano-silica in different percentages: 1 wt.%, 3 wt.%, and 5 wt.%. Scratch morphologies clearly indicate the influential role of reinforcement type and ratio on scratch resistance and active modes of deformation. In the unreinforced epoxy, Figure 4a, the scratch track is highly wide and deep and properly reflects the relatively low value of hardness and also the susceptibility to plastic deformation of the material. Due to the unreinforced situation, the penetration that happened is more significant, and the scratch mark is more profound with evident deformation along the edges of the track. It is discernible that by glass fiber reinforcement, the scratch morphology improves progressively with percentage increase in glass fiber as represented in Figure 4b–d. For example, at 1 wt.% glass fiber, scratch track depicts a decrease both in width and depth compared to unreinforced sample; however, localized deformation was recorded. By increasing the glass fiber ratio up to 3 wt.% (Figure 4c), the scratch resistance is further increased, and the track is narrower and shallower. At 5 wt.% glass fiber content (Figure 4d), the scratch morphology of the glass fiber reinforced sample showed the best performance among the tested glass fiber reinforced samples. In this regard, the plastic deformation flow decreased and accordingly the scratch path became more obvious, but some irregularities still existed on the surface due to the limit in fiber distribution. It is evident that nano-silica reinforcement offers superior performance in terms of both scratch resistance and morphology. As shown in Figure 4e (1 wt.% nano-silica), the scratch track is significantly narrower and smoother compared to the unreinforced and glass fiber reinforced samples, indicating higher hardness and more uniform load distribution. As the nano-silica content increases to 3 wt.% (Figure 4f), the scratch path becomes even finer, with reduced plastic deformation and a much more uniform appearance of the surface ahead of the track edges. The sample containing 5 wt.% nano-silica (Figure 4g) demonstrates the best scratch resistance, with minimal scratch track width and depth, a very uniform surface, and reduced plastic deformation. This can be attributed to the enhanced filling effect of the nano-sized particles within the matrix, leading to increased hardness and more effective stress dissipation under scratching conditions. In comparison, unreinforced epoxy shows the poorest scratch morphology, while the epoxy reinforced with 5 wt.% nano-silica exhibits optimal scratch resistance, producing a narrow and shallow scratch track with minimal surface disruption. This improvement emphasizes that nano-silica modification provides superior scratch performance over glass fiber, particularly at higher reinforcement levels.

**Figure 5:**
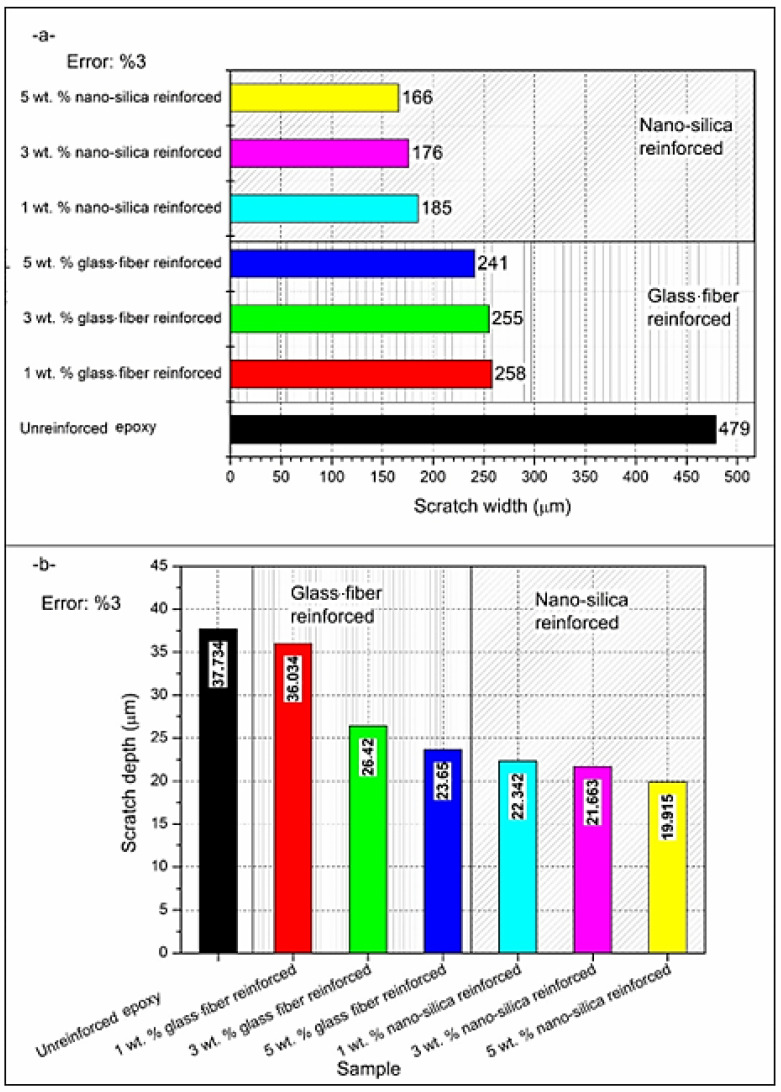
The effect of reinforcement type and ratio on (**a**) scratch width and (**b**) scratch depth.

**Figure 6:**
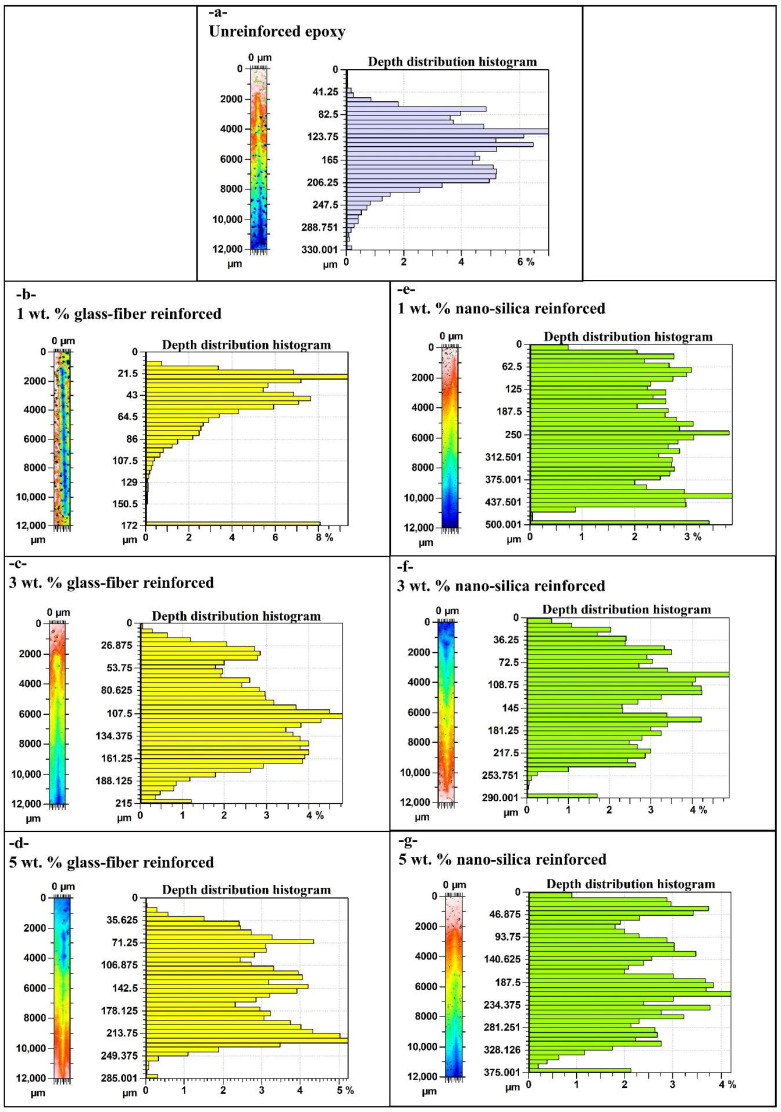
Depth distribution histograms comparison of (**a**) unreinforced, (**b**) 1 wt.% glass fiber reinforced, (**c**) 3 wt.% glass fiber reinforced, (**d**) 5 wt.% glass fiber reinforced, (**e**) 1 wt.% nano-silica reinforced, (**f**) 3 wt.% nano-silica reinforced, and (**g**) 5 wt.% nano-silica reinforced epoxies.

Figure 5a,b illustrates the variations in scratch width and depth of the epoxy as a reinforcement type (glass fiber or nano-silica) and reinforcement percentage (1 wt.%, 3 wt.%, and 5 wt.%). Figure 5a shows that, in the case of nano-silica reinforcement, the scratch width is significantly lower than that of both the unreinforced and glass fiber-reinforced epoxy samples. The scratch width for the unreinforced epoxy was the largest at 479 µm, indicating that it was more susceptible to surface deformation under load. In contrast, all nano-silica reinforced samples exhibit reduced scratch widths as the reinforcement percentage increases. The width decreased to 185 µm at 1 wt.%, 176 µm at 3 wt.%, and reached 166 µm at 5 wt.% nano-silica. This improvement was attributed to the uniform dispersion of nano-silica particles, which increases the hardness and limits plastic deformation of the epoxy. Although glass fiber reinforcement also reduced scratch width compared to unreinforced epoxy, its effect was less pronounced than that of nano-silica. The scratch width for 1 wt.% glass fiber is 258 µm, further reduced to 255 µm at 3 wt.% and 241 µm at 5 wt.%, indicating a more moderate improvement in scratch resistance. For Figure 5b, ‘scratch depth’ denotes the mean of the three baseline-leveled peak depths taken from cross-sections at the start, middle, and end of each scratch; Figure 6 complements this by reporting the full depth distribution along the track and its maximum. Consistent with Figure 5b, scratch depth declines from 37.73 µm in the neat epoxy to 29 µm with chopped glass fiber and to 19.9–21 µm at 5 wt.% nano-silica, indicating that nano-silica more effectively suppresses plowing and subsurface plastic deformation than glass fiber across the studied loadings.

The histograms of depth distribution obtained from scratch tracks across the entire surface for epoxy samples are presented in Figure 6. These histograms reflect variability in depth profiles according to the type and ratio of reinforcement. Differences can be observed clearly between the unreinforced and reinforced epoxy samples. In the case of unreinforced epoxy (Figure 6a), the depth distribution is widely spread across a broad range. The highest depth recorded for unreinforced epoxy was approximately 330 µm; however, the histogram reveals that the majority of the scratch area falls within a depth range of 165 µm to 288 µm. This indicates significant surface deformation, as this material lacks reinforcement. The deep penetration observed suggests that the material has relatively low hardness and resistance to scratch induced deformation.

It can be inferred that there is a marked decrease in maximum scratch depth as the reinforcement percentage increases, as shown in Figure 6b–d for glass fiber reinforcement. For 1 wt.% glass fiber (Figure 6b), the maximum depth decreases to approximately 172 µm, with the majority of depths falling within the range of 43 µm to 129 µm. This improvement demonstrates that even a small amount of glass fiber reduces the material’s tendency toward deep scratches. With the addition of 3 wt.% glass fiber (Figure 6c), the maximum depth further decreases to around 215 µm, with the majority of depths falling between 53 µm and 161 µm. Finally, for 5 wt.% glass fiber reinforcement (Figure 6d), the histogram shows a more even distribution of depth in the range of 35 µm to 249 µm, with the maximum depth around 285 µm, indicating a reduction in deeper deformations. Although glass fiber reinforcement reduces deep scratches, it does not completely eliminate them, likely due to possible agglomeration of fibers and less uniform distribution.

Nano-silica reinforcement demonstrates superior performance compared to glass fiber in terms of reducing scratch depth (Figure 6e–g). For 1 wt.% nano-silica (Figure 6e), the histogram shows a maximum depth of about 437 µm, but the majority of the scratch depth lies between 0 µm and 437 µm, representing an improvement compared to the unreinforced sample. For 3 wt.% nano-silica (Figure 6f), the maximum depth was reduced to around 290 µm, with most of the scratch area falling between 36 µm and 253 µm, further demonstrating a reduction in scratch depth. The most significant improvement was observed at 5 wt.% nano-silica (Figure 6g), where the histogram shows a maximum depth of 375 µm, and a large part of the scratch area lies within the range of 93 µm to 281 µm. This substantial reduction in depth distribution highlights the ability of nano-silica to improve hardness, better distribute the applied load on the scratch surface, and reduce deformation.

**Figure 7:**
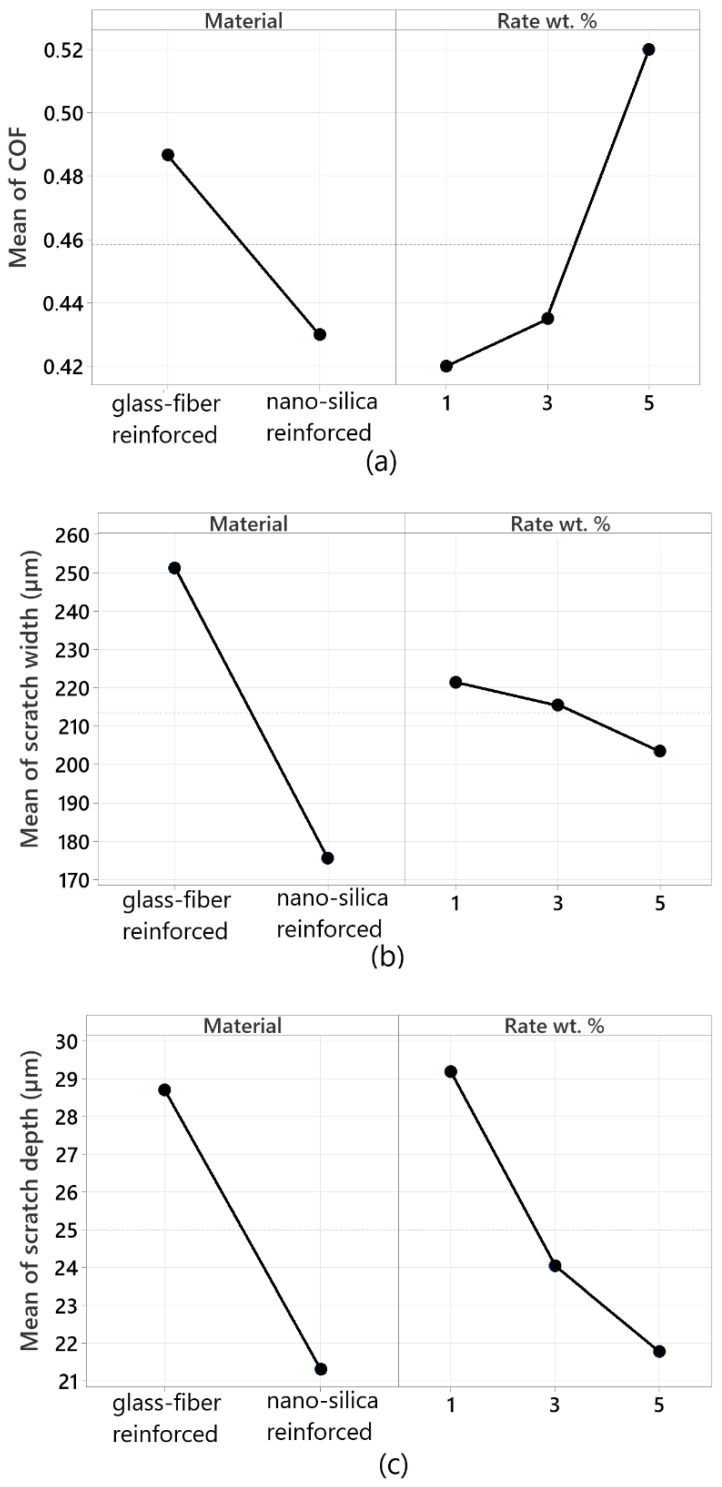
Average effects of glass fiber and nano-silica reinforcement on the (**a**) COF, (**b**) scratch width, and (**c**) scratch depth of epoxy resin.

In general, unreinforced epoxy exhibits the widest and deepest scratch distribution, while glass fiber and nano-silica reinforcements improve scratch resistance. While Figure 6e exhibits the widest tail and the deepest point, the depth value marked 500.001 µm corresponds to a z-range saturation caused by a localized chip-out at the groove boundary; this single clipped pixel inflates the extreme tail but does not change the median trends. Using robust descriptors (median and 90th percentile), both reinforcements still show shallower and tighter scratch-depth distributions than the unreinforced epoxy. Nano-silica, in particular, provides a more homogeneous and effective reduction in depth as the content increases. The histograms confirm that nano-silica reinforcement, especially at 5 wt.%, delivers the best performance in limiting scratch depth and providing a more uniform depth profile along the scratch track. While Figure 6b shows that 1 wt.% glass fiber attains the shallowest single-track maximum depth (172–173 µm), our ‘best performance’ designation for 5 wt.% nano-silica reflects an integrated damage metric—narrower scratch width (166 µm vs. 258 µm; Figure 5a), lower mean depth (21 µm vs. 29 µm; Figure 7c), and a more uniform, repeatable depth profile along the track—by which nano-silica consistently minimizes overall areal damage.

This agrees well with observations recorded within different research studies. Reinforcements like glass fibers and nano-silica have a dramatic effect on the mechanical properties, especially scratch resistance, of epoxy-based composites. Glass fiber reinforcement enhances scratch resistance through reducing the maximum scratch depth while effectively distributing the applied load across the composite. For instance, a study carried out by Mohammed et al. [31] showed that glass fibers improve mechanical properties such as tensile and impact strength, which could, in turn, be responsible for better resistance to surface deformation. In this direction, it was demonstrated in Ref [32] that hybrid reinforcement improves impact resistance, which is again associated with reduced scratch susceptibility. Nano-silica energy performs even better in scratch depth reduction with the improvement in hardness and distribution of applied stress uniformly. In a similar study [33], it was stated that the addition of nano- and micro-fillers to epoxy composites led to improvements in load transfer and interface adhesion and improved wear resistance.

This result agrees with the observation obtained when nano-silica reduces the amount of deformation under scratch testing. Mohanty et al. [34] found that composites exhibited better tribological behavior and reduced wear rates, thus providing superior scratch resistance. All these results indicate that the type and concentration of reinforcement are critical in the scratch performance of epoxy composites. This fact agrees well with the trends described for the histogram of the depth distribution.

### 3.3. Factorial Design and ANOVA Analyses

Factorial design is a widely used experimental method in academic research, where multiple factors are varied simultaneously to study their effects. This approach allows researchers to analyze the main effects of each factor as well as their interactions, making it possible to understand how different factors interact in complex real processes. Factorial design is efficient in terms of both time and cost, as multiple factors can be studied concurrently. One of its major advantages is its ability to detect factor interactions, which enhances the generalization of experimental results. The results from factorial design are more reliable and comprehensive in multifactor systems compared to other methods. Additionally, factorial designs provide flexibility in experimental planning, allowing researchers to optimize parameters and identify critical factors affecting outcomes. For example, in material science, factorial experiments are instrumental in optimizing mechanical properties by analyzing the combined effects of variables such as temperature, pressure, and material composition [35]. Similarly, in engineering, factorial designs have been applied to improve the properties of electrodeposited metals, demonstrating the advantage of full factorial designs over randomized approaches in optimizing material characteristics [36]. This robust approach ensures that the results are not only statistically significant but also practically applicable, providing valuable insights across various disciplines [37].

Table 2 presents the results of a factorial design variance analysis for the COF, scratch width, and scratch depth. In this analysis, the primary focus was not on the *p*-value but on the contribution and effect of each factor. The model accounts for 100% of the variance in COF, indicating that all key factors and interactions are comprehensively addressed. Linear effects explain 67.19% of the total variance, suggesting that the main factors have a significant influence on COF. The material factor accounts for 19.67%, showing that material type has a notable effect on COF. The most influential factor in this model is the weight percentage (Rate wt.%), contributing 47.52%, indicating its strong impact on COF. Two-way interactions, particularly between material and weight percentage (Material*Rate wt.%), contribute 32.81%, signifying that the combination of material type and weight percentage significantly affects COF. These interactions demonstrate that changes in material type and weight percentage will substantially alter COF. Thus, although the F and *p*-values are not critical, it is evident that both weight percentage and material type significantly influence COF. This analysis is essential for understanding the effects of these factors on COF.

In the analysis of scratch width presented in Table 2, the material factor accounts for 96.13% of the variance, indicating that material type has a highly significant impact on scratch width. The weight percentage factor (Rate wt.%) accounts for 3.76% of the variance, suggesting that its influence on scratch width is less pronounced than that of the material. Two-way interactions (Material*Rate wt.%) account for a minimal variance of 0.10%, indicating that the combination of material and weight percentage does not significantly interact to affect scratch width. Therefore, material type is the primary factor influencing scratch width, with a statistically significant effect. While weight percentage plays a relatively minor role, it should still be considered. This analysis emphasizes the importance of optimizing material selection to minimize scratch width.

Regarding scratch depth, Table 2 shows that the material factor accounts for 48.35% of the variance, demonstrating its significant influence on scratch depth. The weight percentage factor (Rate wt.%) accounts for 33.97% of the variance, suggesting that weight percentage also significantly affects scratch depth, albeit to a lesser extent than material type. Two-way interactions (Material*Rate wt.%) contribute 17.69% of the variance, indicating a substantial interaction between material and weight percentage on scratch depth. This interaction emphasizes the combined effect of material and weight percentage in determining scratch depth. The material factor remains the primary influence on scratch width, and its effect is statistically significant. The weight percentage factor contributes notably, and the interaction between these factors produces a significant effect on scratch depth. This analysis highlights the importance of optimizing both material selection and weight percentage to minimize scratch depth.

In the scratch test, the COF value of the unreinforced epoxy resin was determined to be 0.45, while the scratch depth and scratch width were measured at 37.734 µm and 479 µm, respectively. These results clearly highlight the improvement in mechanical performance for the reinforced epoxy samples compared to the unreinforced epoxy. With this data as a baseline, each graph was further analyzed.

Figure 7a below presents the mean effect plot for the average COF. The unreinforced epoxy resin has a COF of 0.45. In comparison, the glass fiber-reinforced resin exhibits a higher COF of 0.48, which may be attributed to the glass fibers creating more friction on the surface. The introduction of nano-silica reinforcement reduced the COF below the reference resin value, lowering it to 0.42 and decreasing the shear resistance of the surface. However, a nano-silica ratio of 5% resulted in a significant increase in COF, suggesting that a higher filler ratio enhances surface interaction.

Figure 7b displays the mean effect plot for the average scratch width. The scratch width of the unreinforced epoxy resin is approximately 479 µm. Adding glass fiber reinforcement slightly improved scratch resistance, reducing the scratch width to around 250 µm. The addition of nano-silica further reduced this width to approximately 170 µm. As the nano-silica ratio increased, the scratch width gradually decreased, reaching its minimum at 5 wt.%. The particle size and distribution of nano-silica significantly improved the abrasive resistance properties of the resin.

Figure 7c shows the mean effect plot for the average scratch depth. The scratch depth of the unreinforced epoxy resin was 37.734 µm, indicating that the material scratches easily. Adding glass fiber reinforcement reduced the scratch depth to 29 µm, demonstrating that the glass fibers increased the material’s hardness and resistance to scratches. The addition of nano-silica reinforcement further reduced the scratch depth to approximately 21 µm, and the depth continued to decrease as the nano-silica ratio increased. Nano-silica significantly enhances the hardness properties of the epoxy resin, improving surface resistance, with this enhancement dependent on particle size.

In conclusion, the introduction of glass fiber and nano-silica reinforcements into the epoxy resin led to substantial improvements in mechanical properties compared to the reference epoxy. The most significant enhancements were observed in scratch width and depth, while the largest decrease in the friction coefficient was noted with nano-silica reinforcement. These test results confirm that nano-silica is an efficient additive for improving abrasive resistance and surface performance. Hence, the results of this work imply that the addition of nano-silica to epoxy composites is highly advantageous for certain applications, such as in automotive coatings, where scratch resistance is of paramount importance combined with a low COF. The resulting reduced wear and enhanced load-carrying capacities could result in improved serviceability of these materials whenever operational demands are placed upon them.

### 3.4. SEM Results

**Figure 8:**
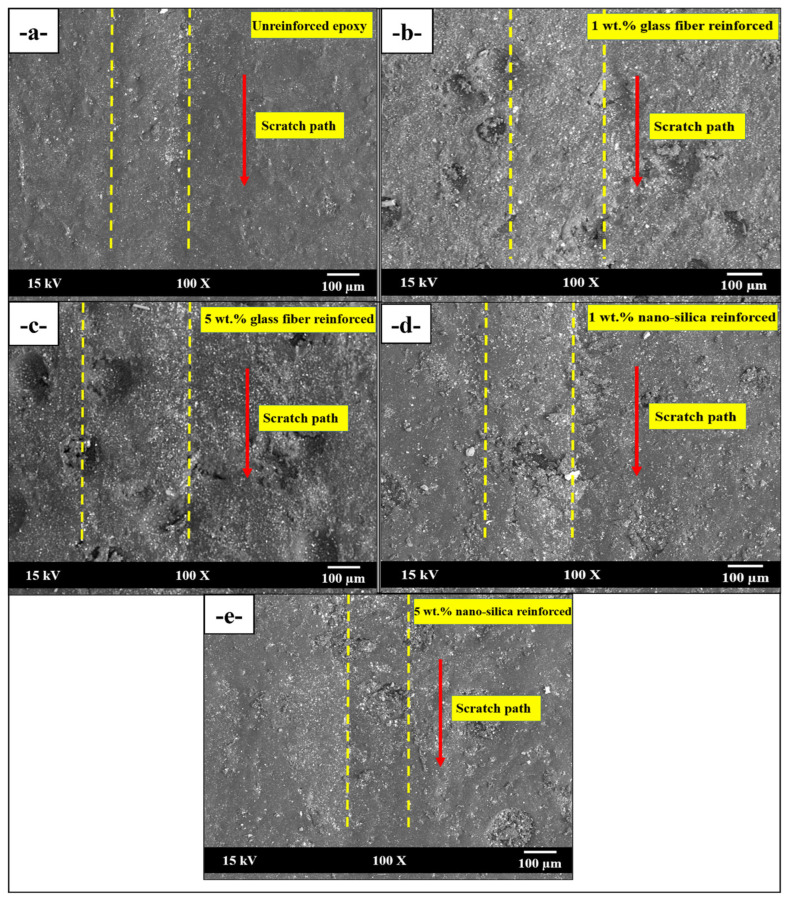
Scratch track SEM images of (**a**) unreinforced, (**b**) 1 wt.% glass fiber reinforced, (**c**) 5 wt.% glass fiber reinforced, (**d**) 1 wt.% nano-silica reinforced, and (**e**) 5 wt.% nano-silica reinforced epoxies.

In the SEM micrograph of the unreinforced epoxy sample (Figure 8a), the severe surface deformation observed along the scratch path is the primary reason for the high COF. In this case, since the epoxy resin did not contain any reinforcing material, the load is entirely carried by the matrix, which increases the frictional resistance between the indenter and the surface during scratching. In Figure 8b, with the addition of 1 wt.% glass fiber, localized hardening due to the fibers partially restricted the surface deformation, which is reflected as a slight decrease in COF values. The glass fibers distributed the scratch load more uniformly within the matrix, thereby reducing the plastic deformation on the surface. However, 5 wt.% glass fiber reinforced sample (Figure 8c), fiber aggregation, and surface irregularities were observed due to the higher reinforcement content. This condition led to direct contact between the fibers and the indenter during scratching, thereby increasing the frictional resistance and causing the COF to rise again.

On the other hand, in the case of nano-silica reinforcement, the effect was less pronounced in SEM images due to the nanoscale size of the particles compared to glass fibers. As shown in Figure 8d,e, corresponding to 1 wt.% and 5 wt.% nano-silica reinforcement, smoother surfaces were obtained relative to glass fiber reinforcement. The silica nanoparticles filled the voids within the matrix, improving surface hardness and homogeneity. A harder and smoother surface reduced the energy loss caused by friction during scratching.

In conclusion, the SEM observations support the COF test results, demonstrating that both glass fiber and nano-silica reinforcements at low concentrations enhance the surface hardness and homogeneity of the epoxy matrix, leading to a reduction in COF. However, at higher reinforcement levels, particularly with glass fiber, fiber agglomeration introduced surface irregularities that increased frictional resistance. SEM observations of the scratched epoxy composites revealed distinct mechanisms for the two reinforcement types. Glass fibers restricted material plowing and stabilized scratch grooves, maintaining surface integrity along the scratch path. Nano-silica dissipated stress and reduced plastic deformation. The measured scratch depth, width, and COF values quantitatively confirmed these trends.

Similar behaviors regarding filler type and size have been reported in PEEK and UHMWPE composites [38,39,40], where nano- and micro-scale reinforcements influenced scratch morphology, plowing patterns, and wear debris formation. For instance, PEEK nanocomposites formed a coherent transfer film that reduced plastic deformation and improved tribological performance, while UHMWPE reinforced with ZnO and Ag showed altered surface roughness, smaller wear debris, and improved wear resistance. These findings provide a context for understanding how filler scale and type can affect scratch behavior, complementing the observations in the epoxy composites.

## 4. Conclusions

The present study makes a significant contribution to the literature on polymer composites, specifically regarding glass fibers and nano-silica reinforcements, by demonstrating that tribological properties are influenced by both the type and the ratio of the reinforcement material used in epoxy resins. The paper highlights the effectiveness of nano-silica as an ideal reinforcement for enhancing scratch resistance, frictional characteristics, and mechanical integrity in epoxy-based materials. This study confirms the positive impact of nano-silica dispersion and its ability to improve load-bearing capacity in the matrix, as evidenced by the reduction in scratch width from 479 µm to 166 µm at a 5 wt.% nano-silica ratio and a decrease in scratch depth from 37.734 µm to 19.915 µm.

The statistical methods employed, such as the Taguchi design and ANOVA, validate the interaction effects between reinforcement type and weight percentage on material performance, providing a solid foundation for future optimization in composite design. From a practical standpoint, this study emphasizes the suitability of nano-silica reinforced epoxies in applications requiring low COF and high scratch resistance, such as in the automotive, aerospace, and electronics sectors. This reduced wear and improved load-carrying capability may contribute to extended service life in such demanding conditions of operation.

Future studies could explore hybrid reinforcement strategies, combining nano-silica with other additives at both the nano- and micro-scale, to achieve synergistic effects that further enhance scratch and wear performance. Additionally, research on the shape, distribution, and functionalization of nano-silica could provide more targeted guidance for optimizing composite properties and behavior.

In conclusion, this study exhibits the groundwork for further research aimed at developing improved epoxy-based composites through specific reinforcement strategies, offering valuable insights into their potential for use in demanding industrial and commercial applications.

## Figures and Tables

**Table 1:** Properties of epoxy resin.

Property	Araldite 2015
Tensile strength (MPa)	31
Tensile modulus (MPa)	1600
Elongation at break (%)	4.2
Viscosity at 25 °C	Thixotropic
Density (g/cm^3^)	1.4
Hardness (Shore D)	75

**Table 2:** Factorial design analysis of variance results for COF, scratch width, and scratch depth.

	Source	DF	Seq SS	Contribution	Adj SS	Adj MS	F-Value	*p*-Value
COF	Model	5	0.024483	100.00%	0.024483	0.004897		
	Linear	3	0.01645	67.19%	0.01645	0.005483		
	Material	1	0.004817	19.67%	0.004817	0.004817	1.2	0.388
	Rate wt.%	2	0.011633	47.52%	0.011633	0.005817	1.45	0.408
	2-Way Interactions	2	0.008033	32.81%	0.008033	0.004017		
	Material*Rate wt.%	2	0.008033	32.81%	0.008033	0.004017		
	Error	0						
	Total	5	0.024483	100.00%				
Scratch width	Model	5	8933.5	100.00%	8933.5	1786.7		
	Linear	3	8924.17	99.90%	8924.17	2974.72		
	Material	1	8588.17	96.13%	8588.17	8588.17	1840.32	0.001
	Rate wt.%	2	336	3.76%	336	168	36	0.27
	2-Way Interactions	2	9.33	0.10%	9.33	4.67		
	Material*Rate wt.%	2	9.33	0.10%	9.33	4.67		
	Error	0						
	Total	5	8933.5	100.00%				
Scratch depth	Model	5	169.65	100.00%	169.65	33.93		
	Linear	3	139.64	82.31%	139.64	46.55		
	Material	1	82.02	48.35%	82.02	82.02	5.42	0.144
	Rate wt.%	2	57.62	33.97%	57.62	28.81	1.92	0.342
	2-Way Interactions	2	30	17.69%	30	15		
	Material*Rate wt.%	2	30	17.69%	30	15		
	Error	0						
	Total	5	169.65	100.00%				

## Data Availability

The datasets presented in this article are not readily available because the data are part of an ongoing study. Requests to access the datasets should be directed to the corresponding author.

## Abbreviations

The following abbreviations are used in this manuscript:

SEM	Scanning Electron Microscopy
ANOVA	Analysis of Variance
